# The predominance of *Penicillium*, *Mucor*, and *Yarrowia* among spoilage fungi in cultured dairy products produced by 3 manufacturers, as revealed by amplicon sequencing

**DOI:** 10.3168/jdsc.2025-0796

**Published:** 2025-09-10

**Authors:** Xiaoxuan Shi, Katerina Roth, Abigail B. Snyder

**Affiliations:** Department of Food Science, Cornell University, Ithaca, NY 14853

## Abstract

Fungal spoilage in cultured dairy products causes consumer dissatisfaction, food waste, and financial losses. This study aimed to identify and characterize fungi responsible for spoilage in cultured dairy products by amplifying the ITS1–5.8S–ITS2 ribosomal DNA (rDNA) region. A total of 200 isolates collected from 154 spoiled dairy products, including yogurt, cottage cheese, and sour cream. The most common genera identified were *Penicillium* (46.5% of isolates), *Mucor* (15% of isolates), and *Yarrowia* (12.5% of isolates). *Penicillium* remained the most frequently isolated fungal genus regardless of the inclusion of preservatives in the dairy products. The internal transcribed spacer (ITS) sequences from this collection and ITS sequences from an additional 483 fungal isolates from cultured dairy downloaded from FoodMicrobeTracker were aligned to assess sequence diversity in their 5.8S rRNA regions. All isolates in more than half (9/15) of the genera did not differ by an SNP in their highly conserved 5.8S region. However, the aligned 5.8S rDNA region from 182 *Penicillium* isolates revealed 5 unique SNPs, and those from 37 *Mucor* isolates had 11 SNP differences. The most sequence diversity was observed in *Candida* with 36 SNPs among 78 aligned 5.8S rDNA regions, demonstrating that variation within this region varies depending on the genus.

## Highlights

• Fungi cause spoilage in cultured dairy products, with or without preservatives.
• *Penicillium, Mucor*, and *Yarrowia* were most commonly isolated and identified.
• Internal transcribed spacer sequencing can be used to identify fungal isolates better than morphology alone.
• Amount of variation between 5.8s rDNA subregions depends on the genus.

## Graphical Abstract

**Figure:**
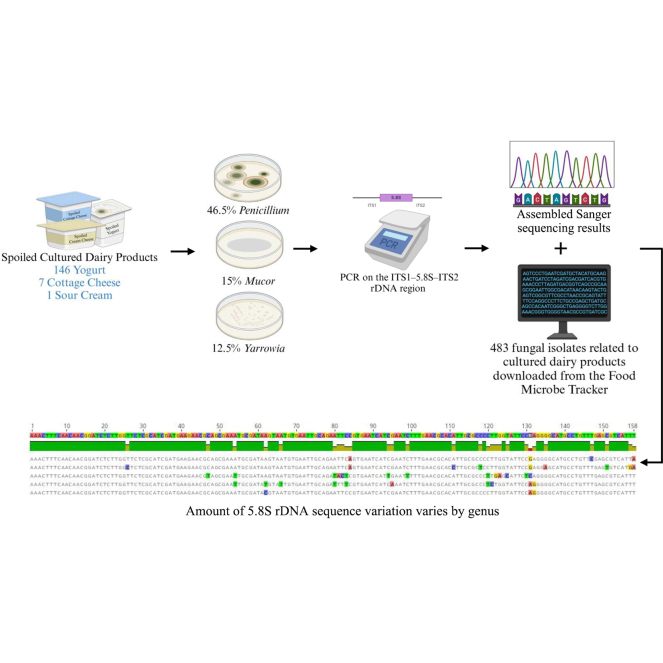
**Summary:** Spoiled cultured dairy products (yogurt, cottage cheese, and sour cream) were collected and plated to isolate fungal cultures. We extracted DNA from the fungi, and PCR was performed on the ITS1-5.8S-ITS2 ribosomal DNA (rDNA) region. The Sanger sequencing results were used to identify the genera to which the fungal isolates belonged. The 3 most common genera identified were *Penicillium, Mucor*, and *Yarrowia*. Additionally, 483 fungal isolates related to cultured dairy products were downloaded from FoodMicrobeTracker and combined with the sequences from this study to compare the amount of variation in the 5.8S rDNA subregion.

Fungal spoilage causes global food waste and economic losses. Studies reporting spoilage occurring in cultured dairy products have been published from countries including France (Garnier et al., 2017b), Brazil (Souza et al., 2023), Egypt (Moubasher et al., 2018; Fetouh et al., 2022), and the United States (Buehler et al., 2018). Fungi (i.e., yeast and molds) commonly contaminate cultured dairy products after pasteurization, leading to changes in texture, odor, and appearance (Snyder and Worobo, 2018). Specifically, fungi are a major cause of spoilage in cultured dairy products such as yogurt due to their ability to grow at low pH levels and at refrigeration temperatures. Additionally, some fungi can ferment lactose and sucrose and hydrolyze milk proteins and fats (Batista et al., 2004; Fekete et al., 2008; Zanutto-Elgui et al., 2019). Addressing spoilage and minimizing subsequent food waste is a topic of importance for the dairy industry to advance quality and sustainability goals.

Producers can use a variety of interventions to prevent fungal growth and contamination. Preservatives, including biopreservatives such as protective cultures and chemical preservatives like potassium sorbate, have been widely used in the food industry (Dehghan et al., 2018; Makki et al., 2020). In addition, sanitizers are used to reduce fungi in the processing environment (Bernardi et al., 2018). The inhibitory activity of preservatives and sanitizers vary among fungi (Bernardi et al., 2018, 2019). Fungi also have a wide range of growth optima, which should be considered when formulating products. Producers can take steps to identify and characterize fungi present in their cultured dairy processing environment, including in the finished product and around the production facility, through environmental monitoring. Accurate identification of spoilage fungi allows producers to track trends in fungal contamination within their facility over time in order to develop more targeted elimination strategies within a preventive quality control program.

Identification of spoilage fungi solely by observing macro- and micro-morphology has been the approach adopted historically, but is an ineffective solution for dairy manufacturers who lack taxonomic expertise (Lücking et al., 2020). Morphology-based identification is highly subjective and time-consuming. A sequencing-based approach targeting a conserved region on the genome has been a reliable approach, allowing for more accurate and efficient identification of fungal species. The nuclear ribosomal internal transcribed spacer (**ITS**) region, which includes 2 internal transcribed spacers (ITS1 and ITS2), and the 5.8S rRNA encoding region in between, has been used as a universal DNA barcode marker for fungi for decades (White et al., 1990; Henry et al., 2000). The amount of variation within this region differs among genera. Therefore, we compared sequence diversity using the 5.8S rRNA region. The 2 objectives of this study were (1) to identify fungi in spoiled cultured dairy products, and (2) to assess sequence diversity of fungi related to cultured dairy products.

From October 2022 to June 2023, a total of 154 spoiled cultured dairy products were received from 3 facilities in New York State. This included 146 yogurts, 7 cottage cheeses, and 1 sour cream product. Products were received in their original containers, with previously opened containers sealed with clear tape. Spoilage was identified in the samples by quality managers due to defects such as pink yeast contamination, bloating, visible mold, shelf-life failure, or customer returns. Several samples failed routine fungal testing but had no observable defects. Some samples contained protective cultures (strains *of Lacticaseibacillus rhamnosus* and *Lacticaseibacillus casei*) or potassium sorbate in the formulations.

Each product was mixed in its original container using a sterile spatula, ensuring thorough mixing, including any residue on the lid. Each sample was diluted in PBS and homogenized using a Stomacher 400 Circulator Lab Blender (Seward Technology Centre, West Sussex, UK). The diluted samples were pipetted onto malt extract agar (**MEA**) plates, incubated at 25°C, and monitored daily for 4 d. Colonies showing distinct morphologies were subcultured onto fresh MEA plates and incubated using the same conditions for 4 d to obtain pure cultures. Distinct morphological characteristics were recorded. Pure cultures were scraped from plates with the addition of PBS and were preserved in 25% glycerol stocks at −80°C for future analysis.

We extracted DNA from pure cultures on MEA plates using the DNeasy PowerSoil Pro Kit (Qiagen, Hilden, Germany). Briefly, the incubated pure culture plate was flooded with 2 mL of PBS. Fungal mycelium and spores were scraped off the agar and diluted in PBS using a sterile L-shape spreader (VWR International, Radnor, PA). Next, 400 μL of culture suspension was transferred to a PowerBead tube, and the manufacturer's protocol was followed until the final elution step, where 40 μL of sterile deionized distilled H_2_O was used for the elution instead of 50 μL of C6 solution.

For a 50-μL reaction, 28.5 μL of sterile double-distilled H_2_O, 10 μL of GoTaq Green 5× PCR buffer (Promega), 5 μL of dNTP, 2 μL of MgCl_2_, 1 μL of forward or reverse primer, and 0.5 μL of GoTaq Flexi Polymerase (Promega) were mixed before adding 2-μL DNA sample. All amplifications were carried out in an Applied Biosystems (Foster City, CA) 2720 Thermal Cycler at 95°C for 5 min, then 95°C for 1 min, 56°C for 1 min, and 72°C for 1 min for 35 cycles, followed by final extension at 72°C for 10 min. We used ITS primers in this study to amplify the ITS1–5.8S–ITS2 ribosomal DNA (**rDNA**) region (ITS 5: 5′-GGAAGTAAAAGTCGTAACAAGG-3′, ITS 4: 5′-TCCTCCGCTTATTGATATGC-3′; White et al., 1990). Sanger sequencing was performed at the Cornell Institute for Biotechnology (Ithaca, NY). Sequencing reads were aligned using Geneious Prime version number: 2023.2 (https://www.geneious.com). Taxonomic identification was performed using the using the National Center for Biotechnology Information (**NCBI**) database (Sayers et al., 2025) Basic Local Alignment Search Tool (https://blast.ncbi.nlm.nih.gov/Blast.cgi).

The 24 fungal genera isolated from spoiled cultured dairy products were searched in the FoodMicrobeTracker (**FMT**) database (https://www.foodmicrobetracker.net/). Relevant ITS sequences were collected from this resource to expand the number of sequences used in the downstream sequence diversity analysis. Isolates from cheese, yogurt, fruit preparation, kefir, air sediment, and equipment were included. One *Penicillium* isolate with a sequence length less than 100 bp was excluded from the downstream analysis due to the short sequence length. The database did not include any relevant *Paecilomyces*, *Sporobolomyces*, *Botryotinia*, *Bullera*, *Leucosporidium*, *Neoascochyta*, *Rhodosporidiobolus*, *Pichia*, or *Trametes* isolates. For each genus, the total number of isolates from spoiled cultured dairy products and from FMT were calculated and genera with a total number equaled to or greater than 5 were selected to proceed for sequence diversity assessment.

Sequence diversity measurement was based on the 5.8S rDNA sequence because it is highly conserved across genera with consistent sequence lengths. Sequences of the 5.8S region from type strain species within the genera identified were downloaded from NCBI. These were aligned with ITS sequences downloaded from FMT and from isolates collected in this study using Geneious Prime software to extract 5.8S sequences. One *Yarrowia* sequence was excluded from downstream analysis due to the presence of multiple ambiguous nucleotides from low sequencing quality. The rest of the 5.8S sequences were then imported into DnaSP6 (Rozas et al., 2017) to obtain the number of polymorphic sites and total number of unique SNPs. Sites with alignment gaps (n = 5) were excluded from the sequence diversity measurement.

In total, 200 unique fungal isolates from 154 spoiled cultured dairy products were isolated. This included 146 yogurt products (188 isolates), 7 cottage cheese products (11 isolates), and one sour cream product (2 isolates). Although spoilage is correlated with the identification of these fungi, it is possible that certain spoilage incidents are attributed to only some fungal groups, especially in the case of multiple isolates from one product or the possibility of low-abundance fungi that were not isolated. There were 24 genera identified among the 200 isolates. Isolates were classified into 3 phyla: Ascomycota (15 genera, 157 isolates), Basidiomycota (8 genera, 14 isolates), and Mucoromycota (one genus, 30 isolates). The most frequently isolated genera from each phylum were *Penicillium* (Ascomycota), *Rhodotorula* (Basidiomycota), and *Mucor* (Mucoromycota). Overall, the 3 most abundant fungal genera isolated are *Penicillium* (93 isolates, 46%), *Mucor* (30 isolates, 15%), and *Yarrowia* (25 isolates, 12%). Together they accounted for >70% of all fungi isolated. In contrast, only 6 isolates or fewer were identified from each of the other genera isolated.

Genera that were less frequently isolated in this study can still pose notable risks for spoilage. For example, 5 *Paecilomyces* isolates were identified from flavored yogurts (Table 1). Many members of the *Paecilomyces* genus are heat-resistant molds that can grow at low oxygen levels and in the presence of preservatives (Houbraken et al., 2008). Because *Paecilomyces* is commonly found in soil, it can easily contaminate fruits in the harvest environment. *Paecilomyces* has been associated with thermally processed fruit preserves, which are often used as ingredients in yogurt with fruit inclusions (Tournas, 1994). If the ascospores were from raw milk, they can be activated by pasteurization, causing spoilage in the final product (Tournas, 1994). Other examples include the 2 isolates of *Saccharomyces* and *Kluyveromyces* that were identified. These genera are known to cause noticeable defects of cultured dairy products due to their lactose or galactose-fermenting abilities, which is not common for fungi (Sati and Bisht, 2006). Many of the less frequently isolated genera found in this study are also associated with flavor defects in yogurt spoilage. For example, *Rhodotorula* can produce extracellular proteolytic enzymes, which leads to bitter flavors and loss of texture. Protease-producing strains also occur within *Saccharomyces*, *Hanseniaspora*, and *Candida* (Fleet, 2011). Therefore, though only isolated from a small number of spoiled products in this study, these genera are still important spoilage organisms and should not be overlooked.

**Table 1:** Yeast and molds isolated from spoiled cultured dairy products manufactured by 3 cultured dairy facilities

Product type	Plain/flavored	No. of products	Isolates from products		Fungal genera	No. of isolates
Yogurt	Plain	14	16		*Mucor*	5
					*Yarrowia*^1^	5
					*Penicillium*	3
					*Geotrichum*, *Rhodosporidiobolus*,^1^*Sporobolomyces*^1^	1^2^
	Flavored	132	171		*Penicillium*	86
					*Mucor*	22
					*Yarrowia*^1^	17
					*Aureobasidium*	6
					*Cladosporium*	5
					*Paecilomyces*	5
					*Candida*^1^	4
					*Rhodotorula*^1^	4
					*Geotrichum*	5
					*Cystofilobasidium*,^1^*Hanseniaspora*,^1^*Kluyveromyces*,^1^*Pichia*,^1^*Sporobolomyces*^1^	2^2^
					*Saccharomyces*,^1^*Botryotinia*, *Bullera*,^1^*Filobasidium*,^1^*Neoascochyta*, *Torulaspora*,^1^*Trametes*	1^2^
Cottage cheese	Plain	6	9		*Penicillium*	3
					*Mucor*, *Yarrowia*^1^	2^2^
					*Leucosporidium*,^1^*Pichia*^1^	1^2^
	Flavored	1	2		*Mucor*, *Clavispora*^1^	1^2^
Sour cream	Plain	1	2		*Penicillium*, *Yarrowia*^1^	1^2^
Total		154	200			

1 Indicates fungal genera presented in yeast form when pure culture was isolated.

2 Indicates number of isolates found in each listed genus, respectively.

Previous studies that have characterized the spoilage fungi isolated from cultured dairy products identified similar genera of fungi, though at different frequencies (Garnier et al., 2017b; Souza et al., 2023). In a similar study done in Spain, spoilage fungi were isolated from 473 yogurt samples, including plain, strawberry-flavored, and banana-flavored yogurts from 3 commercial yogurt brands. *Penicillium* and *Candida* were the 2 most frequently identified fungal genera (García and Fernández, 1984). Although *Penicillium* was the most frequently isolated genus in this study, accounting for 46% of all spoilage, *Candida* accounted for only 2%. In a study conducted in Brazil, 577 fungal isolates were collected from 72 yogurt containers from 4 manufacturers. A total of 10 fungal genera were identified, which is notably fewer total genera than identified in this study. Most of the fungi isolated were yeast such as *Debaryomyces*, *Saccharomyces*, *Mrakia*, *Hansenula* (*Pichia*), and *Candida* (Moreira et al., 2001). In contrast, among the 105 isolates Garnier et al. (2017b) collected from French cultured dairy products including yogurt, yogurt drinks, cream cheese, and cheese, 60% were filamentous fungi, with the most abundant genus being *Penicillium*. In summary, although the specific mycobiota composition in spoiled cultured dairy products differs across studies, a smaller subset of commonly identified genera, most notably *Penicillium*, are consistently reported.

Products received in this study contained either protective culture 1, protective culture 2, potassium sorbate, or no protective culture or potassium sorbate. More than half of the products tested (89 products, 57.8%) in this study contained either a protective culture or potassium sorbate. The distribution of spoilage fungi among these products is shown in Table 2 and indicate that fungal spoilage can still occur when the product formulation includes protective cultures and potassium sorbate. Specifically, 50 fungal isolates were collected from 43 products containing protective culture 1; 52 isolates were collected from 33 products with protective culture 2; 15 isolates were collected from 13 products with potassium sorbate added. The other 84 isolates were collected from 65 products that had neither protective cultures nor potassium sorbate. *Penicillium* was the most isolated fungal genus from cultured dairy products regardless of the addition of protective cultures or potassium sorbate. The number of *Penicillium* isolates from products without protective cultures or potassium sorbate (47 isolates) was similar to those containing either protective cultures or potassium sorbate together (46 isolates). However, the facilities enrolled in this study produce a relatively small volume of products that contain neither protective culture nor potassium sorbate. *Penicillium* spp. can convert the inhibitory sorbic acid to the inactive 1,3-pentadiene (Punt et al., 2022). As a result, the growth of the mold is possible once the active inhibitory substance is neutralized. Likewise, some *Mucor* and *Geotrichum* isolates also can metabolize sorbic acid (Plumridge et al., 2010; Stratford et al., 2007). Apart from *Penicillium*, the fungal genera present in products including protective culture 1 differed from those including protective culture 2. *Yarrowia* was the third most abundant genus in this study (25 isolates, 12.4%) and was isolated the most from products containing protective culture 2 (19/25 isolates). This genus is known for its tolerance of chemical preservatives, compared with other genera commonly isolated from contaminated dairy products, including *Cladosporium*, *Galactomyces*, *Mucor*, *Penicillium*, *Candida*, *and Rhodotorula.* The species *Yarrowia lipolytica* has been shown to have the highest resistance to all tested chemical preservatives (Garnier et al., 2017a). The ability to resist or metabolize preservatives likely contributes to the prevalence of *Penicillium*, *Mucor*, and *Yarrowia* in dairy products.

**Table 2:** Identities of fungi isolated from spoiled cultured dairy products with and without addition of protective cultures or sorbate

Item	Total no. of isolates^1^	Isolates from cultured dairy products with:			
		No protective culture or potassium sorbate (n = 65)	Protective culture 1 (n = 43)	Protective culture 2 (n = 33)	Potassium sorbate (n = 13)
*Penicillium*	93	47	31	8	7
*Mucor*	30	10	—	17	3
*Yarrowia*	25	5	—	19	1
*Aureobasidium*	6	1	5	—	—
*Geotrichum*	6	—	—	6	—
*Cladosporium*	5	4	1	—	—
*Paecilomyces*	5	—	5	—	—
*Candida*	4	2	1	—	1
*Rhodotorula*	4	—	4	—	—
*Pichia*	3	2	—	—	1
*Sporobolomyces*	3	2	1	—	—
*Cystofilobasidium*	2	2	—	—	—
*Hanseniaspora*	2	2	—	—	—
*Kluyveromyces*	2	—	—	2	—
*Saccharomyces*	1	1	—	—	—
*Botryotinia*	1	—	1	—	—
*Bullera*	1	1	—	—	—
*Clavispora*	1	1	—	—	—
*Filobasidium*	1	1	—	—	—
*Leucosporidium*	1	—	—	—	1
*Neoascochyta*	1	—	1	—	—
*Rhodosporidiobolus*	1	1	—	—	—
*Torulaspora*	1	—	—	—	1
*Trametes*	1	1	—	—	—
Total	200	83	50	52	15

1 Each genus was isolated only once per product, so the total number of isolates is the same as the number of products containing that genus. For each column, the number of total isolates exceeds the number of dairy products because fungi from more than one genus were sometimes isolated.

The ITS sequences from a total of 666 isolates (183 isolated in this study, 483 from FMT) were included in the sequence comparison presented in Table 3. The average length of the ITS1–5.8S–ITS2 rDNA region across all isolates was 599 bp with an SD of 159 bp. The shortest ITS sequence, 183 bp, was obtained from a *Yarrowia* isolate, and the longest sequence, 1,164 bp, was from a *Mucor* isolate. The variations in ITS region length is consistent with previous literature, which have reported lengths ranging between 300 bp to 1,200 bp (Heeger et al., 2019). The difference in sequence length across all isolates makes categorization difficult to approach, and therefore the conserved 5.8S region was used to assess the number of polymorphic sites where SNPs were observed within genera. The average sequence length for the 5.8S region was 158 bp, with a standard deviation of 3 bp. The 5.8 S region for *Mucor* was the longest among all (167 bp), whereas that for *Clavispora* was the shortest (152 bp). As shown in Table 3, all isolates in more than half (9/15) of the fungal genera identified did not have any sequence variations in their 5.8S region, which is highly conserved. However, the aligned 5.8S rDNA region from 182 *Penicillium* isolates revealed 5 unique SNPs, whereas those from 37 *Mucor* isolates had 11 SNP differences. The most sequence diversity was observed in *Candida*, with 36 SNPs among 78 aligned 5.8S rDNA regions, demonstrating variation within this region varies depended on the genus.

**Table 3:** Summary of ITS sequencing results among 15 fungal genera from cultured dairy products; fungal isolates were obtained in this study, and additional sequences from fungi previously isolated from cultured dairy products and their processing environments were collected from FoodMicrobeTracker (FMT)

Genus	Number of isolates			ITS rDNA			5.8S rDNA		
	Total	This study	FMT database	Mean length ± SD (bp)	Maximum length (bp)	Minimum length (bp)	Sequence length^1^ (bp)	No. of polymorphic sites	Total no. of unique SNP
*Torulaspora*	201	1	200	754 ± 29	803	712	158	0	0
*Penicillium*	182	92	90	572 ± 107	1,076	425	156	5	5
*Candida*	78	4	74	493 ± 75	932	344	158	30	36
*Clavispora*	42	1	41	358 ± 21	366	276	152	0	0
*Yarrowia*	38	23	15	313 ± 49	378	183	153	0	0
*Mucor*	37	30	7	662 ± 140	1,164	555	167	10	11
*Kluyveromyces*	24	2	22	659 ± 38	722	541	158	0	0
*Cladosporium*	16	5	11	540 ± 124	992	473	158	2	3
*Hanseniaspora*	13	2	11	711 ± 67	747	488	158	0	0
*Aureobasidium*	7	6	1	635 ± 188	1,058	515	158	0	0
*Geotrichum*	6	6	0	386 ± 182	780	267	153	0	0
*Rhodotorula*	6	4	2	593 ± 25	607	542	159	3	3
*Filobasidium*	6	1	5	560 ± 30	621	546	158	0	0
*Paecilomyces*	5	5	0	601 ± 7	606	592	157	0	0
*Saccharomyces*	5	1	4	783 ± 35	845	762	157	0	0

1 Length of 5.8S region recorded in Table 3 represents the length for more than 95% of the isolates.

In conclusion, this study contributes to our understanding of fungal distribution in spoiled dairy products. *Penicillium*, *Mucor*, and *Yarrowia* were the most isolated fungi from cultured dairy products regardless of the inclusion of protective cultures or sorbate in product formulation. Amplicon sequencing of the ITS1–5.8S–ITS2 rDNA region revealed high variability in sequence length. This study exemplifies the types of identification and characterization that cultured dairy producers can use to develop targeted elimination strategies against spoilage fungi.
